# Neuroprotective Role of Quercetin against Alpha-Synuclein-Associated Hallmarks in Parkinson’s Disease

**DOI:** 10.2174/1570159X21666221221092250

**Published:** 2023-05-18

**Authors:** Sabya Sachi Das, Niraj Kumar Jha, Saurabh Kumar Jha, Priya Ranjan Prasad Verma, Ghulam Md Ashraf, Sandeep Kumar Singh

**Affiliations:** 1 Department of Pharmaceutical Sciences and Technology, Birla Institute of Technology, Mesra, Ranchi, 835215,Jharkhand, India;; 2 School of Pharmaceutical and Population Health Informatics, DIT University, Dehradun, 248009, Uttarakhand, India;; 3 Department of Biotechnology, School of Engineering & Technology (SET), Sharda University, Greater Noida, 201310, Uttarpradesh, India;; 4 Department of Biotechnology, School of Applied & Life Sciences (SALS), Uttaranchal University, Dehradun, 248007, India;; 5 School of Bioengineering & Biosciences, Lovely Professional University, Phagwara, 144411, Punjab, India;; 6 Department of Biotechnology Engineering and Food Technology, Chandigarh University, Mohali, 140413, Punjab, India;; 7 Department of Medical Laboratory Sciences, College of Health Sciences, and Sharjah Institute for Medical Research, University of Sharjah, Sharjah, 27272, United Arab Emirates

**Keywords:** Quercetin, Parkinson’s disease, neuroprotective effects, α-synuclein protein, α-synuclein-associated molecular hallmarks, antioxidant

## Abstract

Quercetin, a natural antioxidant, exhibits potential neuroprotective effects by efficiently downregulating α-synuclein protein aggregation and associated neurological hallmarks, responsible for the progression of Parkinson’s Disease.

## 1 INTRODUCTION

Parkinson’s disease (PD) is the second most common neurodegenerative disease (NDD) that progresses slowly and is recognized by the accretion of Lewy neurites/bodies [1]. Both Lewy neurites and Lewy bodies are primarily comprised of accumulated α-synuclein (α-Syn) proteins and post-translationally altered protein by-products [1]. PD is majorly related to motor syndromes, including inactive tremors, gait and stability complications, limb stiffness and bradykinesia), and non-motor syndromes (sleep disorders, olfactive injury, mood instabilities, dementia, and autonomic dysfunctions such as dizziness, constipation, and others) [1]. Despite comprehensive research, the therapeutic possibilities for the management of PD are restricted, offering symptomatic relief only and cannot avoid the disease progression completely. Serine 129 (S129), a principal constituent of α-Syn, is commonly related to the cytoskeleton, trafficking of vesicular proteins, and the enzymes associated with the phosphorylation of serine protein [2]. Phosphorylation of S129 contributes to the accumulation of α-Syn, causing homeostasis and amalgamation of synaptic vesicles and neuronal transmission imperfections [2].

In addition, aggregation of α-Syn and by-products leading to amalgamations have been commonly observed in most of the PD patients and in the case of synucleinopathies such as multiple system atrophy, dementia, and other disorders [2]. The vitagene and its products, including heat shock proteins (HSPs), glutathione, and bilirubin, play a crucial role in regulating the nitric oxide (NO) and reactive nitrogen species, leading to potential neuroprotective effects [3]. Bilirubin, an end product of heme metabolism, has shown beneficial effects for the cells when it interacts with NO, owing to the scavenging effects of bile pigments against the toxic NO and RNS [4]. The Vitagenes family also protects various cellular pathways, included in programmed cellular life, against oxidative stress [5]. Polyphenols, due to their natural antioxidant properties, have shown immense potential in the prevention of NDDs as they effectively improve cellular survival pathways like heat-shock responses [3]. Hormesis, a dose-response concept associated with a pre-conditioning effect, exhibits diverse neuroprotective applications against oxidative stress and aging-mediated pathophysiology in NDDs [5]. This phenomenon can be helpful in the development and establishment of new therapeutic strategies for the effective management of several NDDs, including PD.

## 2 POTENTIAL ROLE OF QUERCETIN IN TARGETING PD CAUSING MOLECULAR HALLMARKS

Quercetin (QC) is a naturally occurring flavonoid, mostly found in onions, apples, tea, berries and leaves, that exhibits diverse pharmacological activities, significantly due to its potential antioxidant activities [6]. QC is widely used for treating or preventing the growth and progression of various types of NDDs, including PD [1, 7]. Mitochondria play a crucial role during oxidative stress conditions in various NDDs by guiding neurons to cellular death [8]. Antioxidants act as a potential neuroprotectant by inducing protective actions against oxidative stress to mitochondria, leading to a delay in the onset or progression of NDDs. In addition, the antioxidants significantly reduce the age-associated damage to mitochondrial DNA and oxidative stress in NDDs [8]. QC exhibits a crucial role in targeting PD by enhancing mitochondrial morphology, monitoring atypical protein aggregations, decreasing neuro-degenerative responses, improving memory discrepancy, and protecting the brain against cerebral losses [1].

The effect of QC has shown a promising role in protein folding and aggregation. QC also effectively decreases the fibrillization of α-Syn and potentially prevents the accumulation of tau neurofibrillary tangles (NFTs) and amyloid-β (Aβ) plaques within the brain [1]. α-Syn has been reported as one of the major factors of PD pathogenesis and other NDDs, usually recognized as synucleinopathies [2]. Various studies have investigated QC for its inhibitory effects against α-Syn aggregations and different amyloid proteins. Researchers anticipated that the neuroprotective activities of QC can be accredited to its capability to attach specifically with α-Syn through covalent bonds, resulting in the α-Syn-QC complex [9], which enhances hydrophilicity and avoids fibrillation [10]. Masuda and co-workers reported that QC exhibited potential anti-fibrillization effects and significantly inhibited α-Syn fibrillization [11]. In another study, recombinant α-Syn was incubated alongside QC (5-100 μM) at 37 °C under continual agitation and observed that the QC effectively inhibited α-Syn aggregation with improved anti-fibrillization effects [12].

The neuroprotective effects of QC against drug/chemical-induced neurodegeneration have been well-established. Pogacnik and co-researchers evaluated the neuroprotective effects and permeability of QC, cyanidin-3-glucoside (C3G), and epigallocatechin gallate (EGCG) through BBB. QC, C3G, and EGCG efficiently decreased α-Syn aggregation over the pertinent period [13]. In another study, Khan and co-researchers evaluated the neuroprotective effect of QC against lipopolysaccharide (LP)-induced neurotoxicity in treated adult mice. Administration of QC to the LP-induced mice significantly decreased the triggered gliosis, and different inflammatory mediators hindered inflammations in the hippocampus and cortex [14]. In another study, researchers demonstrated the neuroprotective effects of QC against rotenone (RT)-induced neurotoxicity in SH-SY5Y (neuroblastoma-derived) cells. QC potentially reduced the intracellular reactive oxygen species (ROS) level, recovered mitochondrial membrane potential, and regulated apoptosis as well as autophagy processes. QC also rescued the cellular degradation and nuclei shrinking caused due to RT treatment [15]. QC-loaded nanoemulsions exhibited neuroprotective effects against the PD-induced *Caenorhabditis elegans* (*C. elegans*) model by effectively decreasing α-Syn protein and ROS levels, improving the mitochondrial and fat content, and also the life cycle of the treated nematodes [7]. Pretsch *et al.* reported that compounds like ZnSO_4_, QC, apomorphine, and dexamethasone significantly induced metallothionein (MTN; an endogenic metal detoxifying protein), leading to reduced proteotoxicity of Aβ and α-Syn, expressed in *C. elegans* CL2659 and NL5901 strains and also prolonged the lifespan wild-type strain N2 [16].

Oxidative stress, mostly caused due to imbalance in ROS levels, is one of the crucial factors that induce cerebral impairments and aging in NDDs, including PD [17]. Sriraksa and co-workers reported that oral administration of QC enhanced the cognitive effects in the PD-induced rat model by inhibiting the oxidative stress leading to increased neuronal density. Also, the treated animals showed improved spatial memory [18]. Wang and co-researchers demonstrated the neuroprotective effects of QC in 6-hydroxydopamine (6-HDOPA)-induced male Sprague-Dawley rats of PD and also in 6-HDOPA-treated PC12 (pheochromocytoma) cells. *In vitro* results showed that QC significantly enhanced the improved mitochondrial content, decreased oxidative stress, enhanced the mitophagy markers (Parker and PINK1) level, and simultaneously reduced α-Syn aggregations [19].

## CONCLUSION AND FUTURE PERSPECTIVES

QC has been proven to be one of the most potent natural antioxidants for treating PD and PD-associated neurological processes [20]. Although QC exhibits potent therapeutic effects against PD, its usage as a therapeutic agent has been limited due to poor aqueous solubility and low bioavailability. Moreover, these limitations can be overcome with applications of nanotechnological platforms such as liposomes, polymeric nanoparticles, nanoemulsions, nanocapsules, and others. Nanocarriers can also help in enhancing the permeability of QC through biological barriers in the brain like BBB, leading to improved targetability with no or negligible toxicity [6, 20]. Thus, with pre-clinical and clinical assessments, a QC-encapsulated stable, targeted, and non-toxic delivery system can be established for treating PD and PD-associated neuronal processes.

## LIST OF ABBREVIATIONS

Aβ: Amyloid-β
α-Syn: α-synuclein
BBB: Blood Brain Barrier
EGCG: Epigallocatechin Gallate
HSPs: Heat Shock Proteins
NDDs: Neurodegenerative Disease
NFTs: Neurofibrillary Tangles
NO: Nitric Oxide
PD: Parkinson’s Disease
QC: Quercetin
ROS: Reactive Oxygen Species
